# Association between smoking and all-cause mortality in Parkinson’s disease

**DOI:** 10.1038/s41531-023-00486-0

**Published:** 2023-04-11

**Authors:** Seo Yeon Yoon, You Hyun Park, Sang Chul Lee, Jee Hyun Suh, Seung Nam Yang, Dae Ryong Kang, Yong Wook Kim

**Affiliations:** 1 Department and Research Institute of Rehabilitation Medicine, Yonsei University College of Medicine, Seoul, Republic of Korea; 2 Department of Biostatistics, Yonsei University, Seoul, Korea; 3 Department of Rehabilitation Medicine, College of Medicine, Ewha Womans University, Seoul, Korea; 4 Department of Physical Medicine & Rehabilitation, Korea University Guro Hospital, Seoul, Republic of Korea; 5 Department of Precision Medicine & Biostatistics, Yonsei University Wonju College of Medicine, Wonju, Republic of Korea

**Keywords:** Parkinson's disease, Epidemiology

## Abstract

We aimed to investigate the association between smoking status and all-cause mortality of Parkinson’s disease (PD). Among the whole nationwide population data from Korea National Health Insurance Service, newly diagnosed PD was selected, and all-cause mortality was evaluated. The systematic review was performed through a literature search on the PubMed, EMBASE, and Cochrane Central Register of Controlled Trials databases. Among 26,080 individuals with PD, there was no significant association between smoking status and all-cause mortality in a nationwide cohort study (ex-smoker, HR 0.1.03, 95% CI 0.97–1.10; current smoker, HR 1.06, 95% CI 0.96–1.16). The systematic review, including six prospective cohort studies, also found a nonsignificant association. PD smokers tended to have fewer deaths from neurologic causes but were significantly more likely to die from smoking-related cancers such as lung cancer. We presented a nonsignificant association between smoking and mortality of PD, and cigarette smoking is not recommended in individuals with PD.

## Introduction

Various lifestyle factors have been suggested to be associated with Parkinson’s disease (PD) risk. Although some lifestyle factors, such as alcohol consumption or dietary habits, showed controversial results with PD development, smoking has shown a relatively consistent association with PD^1–3^. Many previous studies have shown that smoking is negatively associated with PD risk^2,4^, and some studies have suggested an inverse dose-response association^5,6^. Therefore, it has been suggested that cigarettes have neuroprotective effects in PD development, and several trials for the treatment of PD using ingredients of cigarettes such as nicotine have been performed^7,8^.

On the other hand, there have been only a few studies on the association between smoking status and mortality in PD^9–11^. In a recent study in 2019, current smoking was not associated with mortality of PD, whereas former smoking was associated with increased mortality of PD^9^. In this study, the authors evaluated the association between various lifestyle factors and PD progression and mortality; thus, detailed information on smoking status in PD was not described. Previous studies were mostly conducted in Western countries with a relatively small number of participants, and detailed information on PD participants according to smoking status was not sufficiently presented^9–11^.

The etiology of PD is multifactorial, including genetic and environmental factors^12,13^. Although the main treatment of PD is pharmacotherapy, lifestyle factors could affect disease progression, and self-management by adopting a positive lifestyle would be beneficial for disease management^14–16^. The neuroprotective effect of smoking in PD development has been suggested in many previous studies; however, whether quitting or maintaining cigarette smoking is helpful for disease management in PD is still unclear^12,17,18^. Therefore, the objective of this study was to investigate the association between smoking status and all-cause mortality in PD using a nationwide population-based cohort in Korea. The Korean National Health Insurance Service (NHIS) provides a biannual National Health Screening Program (NHSP) to all beneficiaries, and smoking status was obtained from the NHSP questionnaire. Additionally, we performed a systematic review of relevant data to clarify the current evidence regarding the association between smoking and mortality in PD.

## Results

### Baseline characteristics of the study population

The baseline characteristics of the study population are shown in Table 1. Among individuals with PD, 21,880 (77.95%), 4653 (16.58%), and 1535 (5.47%) were never-, ex-, and current smokers, respectively. The current smokers were younger and more likely to be male than never-smokers and ex-smokers. The mean age of current smokers was 65.50 years, which was approximately 5 years younger than that of never-smokers. Never smokers showed higher Charlson Comorbidity Index scores and were more likely to have comorbidities than ex- and current smokers. Regarding lifestyle factors, never-smokers were less likely to drink alcohol and engage in regular physical activity than ex- and current smokers. The mean follow-up duration from PD onset to death was 5.60 ± 2.19, 5.19 ± 2.20, and 5.99 ± 2.22 years for never-smokers, ex-smokers, and current smokers, respectively.

**Table 1:** Demographic and medical characteristics of individuals with Parkinson’s disease.

Variables	Total (*n* = 28,068)	Never-smoker (*n* = 21,880)	Ex-smoker (*n* = 4653)	Current smoker (*n* = 1535)	*p-*Value
Age (years)	70.13 ± 9.44	70.64 ± 9.22	69.24 ± 9.42	65.50 ± 11.03	<0.0001^a,b,c^
Sex					
Male	12,243 (43.62)	6418 (29.33)	4461 (95.87)	1364 (88.86)	<0.0001^a,b,c^
Female	15,825 (56.38)	15,462 (70.67)	192 (4.13)	171 (11.14)	
Income levels					
Q1 (lowest)	4970 (17.71)	3811 (17.42)	791 (17.00)	368 (23.97)	<0.0001^b,c^
Q2	3813 (13.58)	2962 (13.54)	612 (13.15)	239 (15.57)	
Q3	6127 (21.83)	4744 (21.68)	1012 (21.75)	371 (24.17)	
Q4 (highest)	13,158 (46.88)	10,363 (47.36)	2238 (48.10)	557 (36.29)	
Residential area (urban)	11,236 (40.03)	8548 (39.07)	2091 (44.94)	597 (38.89)	<0.0001^a,c^
Insurance type					
National health insurance	27,245 (97.07)	21299 (97.34)	4534 (97.44)	1412 (91.99)	<0.0001^b,c^
Medical aid	823 (2.93)	581 (2.66)	119 (2.56)	123 (8.01)	
Charlson comorbidity index	3.76 ± 2.53	3.80 ± 2.53	3.72 ± 2.56	3.36 ± 2.49	<0.0001^b,c^
Charlson comorbidity index (>4)	13,596 (48.44)	10,769 (49.22)	2189 (47.04)	638 (41.56)	<0.0001^a,b,c^
†Hypertension	18,008 (64.16)	14,143 (64.64)	2975 (63.94)	890 (57.98)	<0.0001^b,c^
†Dyslipidemia	12,444 (44.34)	9793 (44.76)	2059 (44.25)	592 (38.57)	<0.0001^b,c^
†Pneumonia	15,320 (54.58)	12,363 (56.50)	2243 (48.21)	714 (46.51)	<0.0001^a,b^
†Osteoporosis	9352 (33.32)	8624 (39.41)	547 (11.76)	181 (11.79)	<0.0001^a,b^
Drinker	3616 (12.92)	1666 (7.69)	1360 (29.30)	590 (38.51)	<0.0001^a,b,c^
Physically active individuals	12,220 (43.54)	8921 (40.77)	2622 (56.35)	677 (44.10)	<0.0001^a,b,c^
Body mass index (kg/m^2^)	23.76 ± 3.34	23.77 ± 3.41	23.83 ± 3.02	23.39 ± 3.16	<0.0001^b,c^
Waist circumference	83.00 ± 14.31	82.40 ± 15.50	85.45 ± 8.53	84.15 ± 8.80	<0.0001^a,b,c^
Systolic blood pressure (mmHg)	126.51 ± 15.92	*126.76* ± *16.00*	126.01 ± 15.70	124.36 ± 15.24	<0.0001^a,b,c^
Diastolic blood pressure (mmHg)	76.30 ± 10.03	76.39 ± 10.01	76.10 ± 10.12	75.68 ± 9.94	0.0091^c^
Laboratory findings					
Total cholesterol (mg/dL)	183.65 ± 40.01	185.31 ± 40.27	176.63 ± 37.94	181.15 ± 40.07	<0.0001^a,b,c^
Hemoglobin	13.22 ± 1.60	12.99 ± 1.53	14.03 ± 1.55	14.12 ± 1.57	<0.0001^a,b^
Fasting glucose	105.94 ± 29.52	105.88 ± 29.66	105.90 ± 27.62	106.92 ± 32.79	0.4075
Levodopa equivalent daily dose					
Initial	22.31 ± 36.36	22.19 ± 36.20	22.47 ± 35.67	23.53 ± 40.34	0.5914
Last follow-up	412.41 ± 464.61	410.63 ± 473.34	431.74 ± 474.24	376.50 ± 247.29	0.0003^a,b,c^

Values are presented as mean ± SD or number (%).

^†^Other co-morbidities are not included in CCI.

*P* for Bonferroni post-hoc analysis <0.015.

^a^Never-smoker vs. ex-smoker.

^b^Never-smoker vs. current smoker.

^c^Ex-smoker vs. current smoker.

### Risk of all-cause mortality of PD by smoking status

**Fig. 1:**
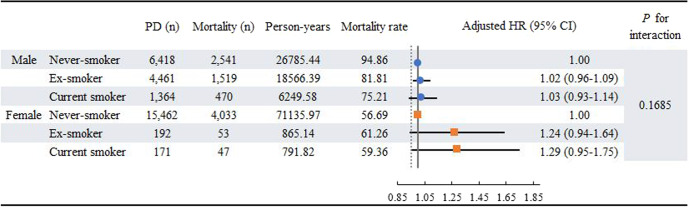
Adjusted hazard ratios on the risk of all-cause mortality in patients with Parkinson’s disease according to smoking status stratified by sex. Association between smoking status and all-cause mortality in Parkinson’s disease was evaluated stratified by sex using Cox proportional hazard regression analysis. In the subgroup analysis by sex, there was no significant association between smoking status and all-cause mortality in either sex.

During the follow-up period (mean 5.54 ± 2.20 years), 8663 deaths occurred in PD patients. The survival distribution according to smoking status in patients with PD, using the log-rank test, is shown in Supplementary Fig. 1. We developed the following three models based on different covariables for all-cause mortality in individuals with PD: Model 1, unadjusted; Model 2, adjusted for age, sex, income level, and residential area; and Model 3, adjusted for age, sex, income level, residential area, comorbidities, lifestyle factors (drinking and physical activity), waist circumference, and LEDD. Compared with never-smokers, the risk of death in PD was not significantly different between ex-smokers (HR 0.97, 95% CI 0.81‒1.03) and current smokers (HR 1.02, 95% CI 0.92‒1.12) when adjusted for sociodemographic variables in Model 2. Similarly, in Model 3, there was no statistically significant association between smoking status and all-cause mortality in patients with PD (ex-smoker, HR 1.03, 95% CI 0.97‒1.10; current smoker, HR 1.06, 95% CI 0.96‒1.16) (Table 2). In the sensitivity analysis of 14,337 patients with PD who maintained the same smoking status in the second NHSP questionnaire, the results were consistent with our main findings (Table 3). In the subgroup analysis by sex, there was no significant association between smoking status and all-cause mortality in either sex (*p* for interaction = 0.1685) (Fig. 1).

**Table 2:** Cox proportional hazard regression analysis on the risk of all-cause mortality in patients with Parkinson’s disease according to smoking status.

	PD (*n*)	Mortality (*n*)	Person-years	Mortality rate	Model 1	*p*-Value	Model 2	*p*-Value	Model 3	*p*-Value
Never-smoker	21,880	6574	97921.41	67.14	1.00		1.00		1.00	
Ex-smoker	4653	1572	19431.54	80.90	1.25 (1.19–1.32)	<0.0001	0.97 (0.91–1.03)	0.2598	1.03 (0.97–1.10)	0.3134
Current smoker	1535	517	7041.41	73.42	1.08 (0.99–1.19)	0.0793	1.02 (0.92–1.12)	0.7595	1.06 (0.96–1.16)	0.2842

The mortality rate is the incidence of mortality per 1000 person-year.

Model 1: unadjusted.

Model 2: adjusted for age, sex, income level, and residential area.

Model 3: adjusted for age, sex, income level, residential area, comorbidities, lifestyle factors (drinking, physical activity), waist circumference, and levodopa equivalent daily dose (last follow-up).

**Table 3:** Cox proportional hazard regression analysis on the risk of all-cause mortality in individuals with Parkinson’s disease with two health screening data according to smoking.

	PD (*n*)	Mortality (*n*)	Person-years	Mortality rate	Model 1	*p*-Value	Model 2	*p*-Value	Model 3	*p*-Value
Never-smoker	11,512	2449	64489.23	37.98	1.00		1.00		1.00	
Ex-smoker	2583	626	13411.40	46.68	1.32 (1.21–1.44)	<0.0001	0.95 (0.86–1.05)	0.3281	0.98 (0.88–1.09)	0.6667
Current smoker	242	140	3247.09	43.12	1.01 (0.85–1.20)	0.9127	0.92 (0.77–1.10)	0.3817	0.93 (0.77–1.12)	0.4309

The mortality rate is the incidence of mortality per 1000 person-year.

Model 1: unadjusted.

Model 2: adjusted for age, sex, income level, and residential area.

Model 3: adjusted for age, sex, income level, residential area, comorbidities, lifestyle factors (drinking, physical activity), waist circumference, and levodopa equivalent daily dose (last follow-up).

### Risk of all-cause mortality of patients with PD by smoking duration and intensity

In the analyses of the association between the duration and intensity of smoking and mortality, PD current smokers with ≥10 pack-years or 10 years of smoking duration showed reduced mortality, whereas those with <10 pack-years or 10 years of smoking duration showed increased mortality, and this association was not significant in PD ex-smokers. Overall, however, there was no statistically significant association between pack-years (*p* for interaction = 0.7695), duration (*p* for interaction = 0.7015), and intensity (*p* for interaction = 0.8459) of smoking and all-cause mortality in PD (Supplementary Table 1).

### Cause of death by smoking status

**Fig. 2:**
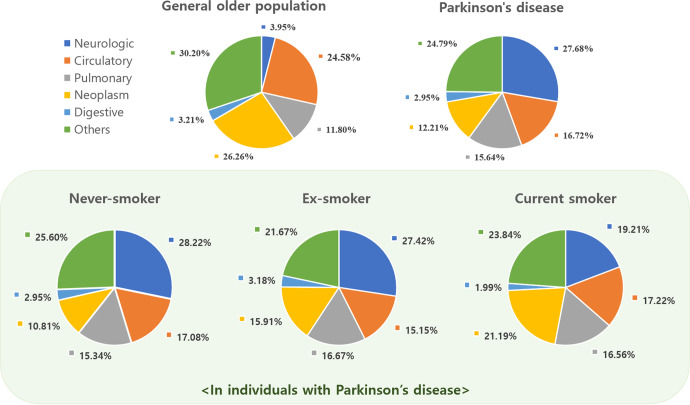
Cause of mortality in general elderly populations and Parkinson’s disease according to smoking status. PD patients died more from neurological and pulmonary causes than the general older population. According to smoking status in individual with PD, current smokers tended to have fewer death from neurological origins than never- and ex-smokers, but death from neoplasm were significantly higher than never smokers.

The causes of death in the general older population (≥60 years) and PD participants are shown in Fig. 2. The causes of death in the general older population were obtained from the Korea National Statistical Office. PD participants died more from neurological and pulmonary causes than the general older population. Common diseases according to each cause of death in PD are as follows: PD for neurologic causes, myocardial infarction, stroke, and heart failure for circulatory causes, pneumonia for pulmonary causes, gastrointestinal bleeding, ileus, and intestinal obstruction for digestive causes, and lung cancer for neoplasms. According to smoking status in individuals with PD, current smokers tended to have fewer death from neurological origins than never- and ex-smokers (*p* = 0.0572), but death from neoplasm were significantly higher than never smokers (Supplementary Table 2). The proportion of deaths from smoking-related cancers, such as lung cancer, was significantly different across the three groups (data not shown): never smokers, 1.55%; ex-smokers, 3.33%; current smokers, 7.95% (*p* < 0.0001).

### Systematic literature review and qualitative analysis

A flowchart of the search process is presented in Supplementary Fig. 2. In total, 304 studies were identified during the initial search. After duplicates were removed, 253 studies remained. Based on the screening of titles and abstracts, 245 studies were discarded. The full texts of the eight remaining papers^9–11,19–23^ were assessed in more detail for eligibility, and two studies with insufficient outcomes were excluded. Finally, 6 studies^9–11,19,20,22^, including 1796 PD participants, were included in the qualitative analysis.

Table 4 summarizes the characteristics of the included studies. All six studies were prospective cohort studies performed in Western countries. The number of PD participants in the included studies ranged from 150 to 560^19,22^. Four studies included participants of both sexes^9,10,20,22^, whereas the other two studies included only male PD patients^11,19^. The majority of studies included a control group, compared the mortality rate with PD, and further evaluated the association between smoking and mortality in PD^9–11,19,20^. There was clinical heterogeneity regarding the objective and study design across the studies. Two studies in the 2000s focused on the association between smoking status and mortality in PD^10,11^. On the other hand, smoking status was used as one of the risk factors for mortality in PD in two studies^9,19^ and only as an adjusting variable for mortality in PD in two studies^20,22^. Thus, detailed information on mortality according to smoking status in PD participants was difficult to obtain. Overall, there was no significant association between smoking status and mortality in PD in the six studies of systematic literature review studies.

**Table 4:** Summary of studies included in the systematic review.

Author	Year	Country	Design of study	Sex	Case (*n*)	PD smoker (*n*)	PD non-smoker (*n*)	Control (*n*)	Median follow-up duration (y)	Death in PD	Results
^a^Alves et al.^10^	2004	Norway	Prospective cohort	Both	239	78	161	200	8 (max)	138	RR = 1.06, *p* = 0.75
^a^Chen et al.^11^	2006	USA	Prospective cohort	Male	288	118	170	51,012	6.7	92	RR = 1.00, *p* = 0.95
^b^Driver et al^22^.	2008	USA	Prospective cohort	Male	560	273	287	560	5.8	200	*p* for interaction = 0.87
^b^Pail et al.^9^	2019	USA	Prospective cohort	Both	360	–	–	341	5.3	209	HR = 1.90 (0.84–4.30), *p* = 0.12
^c^Schneider Medeiros et al.^25^	2020	Brazil	Prospective cohort	Both	150	41	109	-	8.9	62	HR = 1.17 (0.63−2.17), *p* = 0.62
^c^Gonzalez et al.^23^	2022	Norway	Prospective cohort	Both	190	-	-	203	11 (max)	65	HR = 1.00 (0.98−1.02), *p* = 0.67

^a^Studies focusing on the association between smoking status and mortality in PD.

^b^Studies where smoking status was used as one of the risk factors for morality in PD.

^c^Studies where smoking status was used as an adjusting variable for morality in PD.

## Discussion

In this nationwide population-based study, including 28,060 PD participants, we investigated the association between smoking status and all-cause mortality in patients with PD. Our results suggest that there was no significant association between smoking status, including pack-years, duration, intensity, and all-cause mortality, in patients with PD. A systematic review including six prospective cohort studies also found a nonsignificant association between smoking and mortality in patients with PD. Subgroup analysis by sex and sensitivity analysis, considering the maintenance of smoking status, showed consistent results. The causes of death in patients with PD according to smoking status were significantly different between the groups. PD smokers tended to have fewer deaths from neurologic causes but were significantly more likely to die from smoking-related cancers, such as lung cancer. Based on our results, cigarette smoking for its neuroprotective effects is not recommended in individuals with PD.

Many studies have shown an inverse association between smoking and PD risk, and several possible mechanisms have been proposed. The personality trait in PD is one of the explanations^24–26^. The brain dopamine system has been suggested to mediate particular behavioral and personality traits in PD that sometimes precede motor symptoms by decades^25^. Thus, individuals with prodromal PD had low risk-taking personality traits or low impulsive sensation-seeking traits, which leads to less inclination to smoke or more likely to quit^24^. On the other hand, whether cigarette smoking has a real neuroprotective effect on PD development has also been investigated. Since nicotine stimulates dopaminergic neurons and the release of dopamine^27,28^, it has been tried for PD, showing neuroprotective effects in animal models but nonsignificant results in clinical trials^7,8,17,29^. Other ingredients of cigarettes, such as anabasine, cotinine, and hydroquinone, have been scarcely studied, and more research on these biological mechanisms is necessary^4,30^.

Although the exact neuroprotective mechanism of smoking on PD risk is still uncertain, the neuroprotective effect of smoking could also affect disease progression and mortality in PD. However, in our systematic review, we could not find a significant association between smoking status and mortality of PD^9–11,19,20,22^. There have been only six previous studies addressing this issue, and only two studies focused on the relationship between smoking status and mortality in PD^10,11^. Five of the six studies included a comparison group and compared mortality rates with PD and further evaluated the association between smoking and PD^9–11,19,20^. Overall, detailed information about smoking status and related outcomes in PD participants has been described insufficiently, and the reporting methods of results were heterogeneous across the studies, which made it difficult to pool the results to meta-analysis. Through this literature search, we realized the need for more studies focusing on the association between smoking and mortality, with detailed information on smoking and relevant outcomes in PD.

In our cohort study of 28,060 individuals with PD, there was no significant association between smoking and mortality, which is consistent with previous results. Considering the possibility of smoking status changes, we performed a sensitivity analysis on 14,337 PD participants who had maintained the same smoking status on the second NHSP, and the results were still nonsignificant. There were significant differences in baseline characteristics between groups according to smoking status. PD current smokers were younger and more likely to be male than never- and ex-smokers. In regards to LEDD, initial LEDD was not different between groups, but at the last follow-up, current smokers have prescribed significantly lower doses of LEDD than never- and ex-smokers. Therefore, we intensively adjusted these variables using three models. In model 1, there were differences in mortality according to smoking status in individuals with PD. However, in model 2, these differences disappeared as a result of adjusting for age, sex, income level, and residential area, which were significantly different between groups. We investigated which factors influenced the results and found that adjustment for sex rendered the association between smoking status and mortality nonsignificant (data not shown). The male predominance of smokers appears to bias the association between smoking status and PD mortality in model 1.

Recently, sex-related differences in PD, including incidence, clinical symptoms, and outcomes, have been suggested. PD incidence was about 1.5 times higher in males than females^31,32^, and PD males have been shown to be related to a higher mortality rate than PD females^33–35^. Therefore, we conducted a sex-stratified analysis to evaluate whether the association between smoking status and PD mortality was different according to sex. The results also showed no significant association between smoking status and PD mortality in both males and females.

We also investigated the association between the intensity and duration of smoking and mortality in PD. PD current smokers with ≥10 pack-years or 10 years of smoking duration showed reduced mortality, whereas those with <10 pack-years or 10 years of smoking duration showed increased mortality, and this association was not significant in PD ex-smokers. Based on these results, it seems possible that smoking timing or dose (intensity or duration) influenced mortality in individuals with PD. Overall, however, the analyses for the association between the intensity and duration of smoking and PD mortality were not statistically significant. The number of individuals with PD with high intensity or long duration of smoking was relatively small, which requires careful consideration when interpreting the results. Future studies on the neuroprotective effects of smoking in PD according to the duration after quitting are warranted.

Smoking has been suggested to increase mortality, especially due to smoking-related cancers and respiratory causes such as chronic obstructive pulmonary disease^36,37^. However, in our results, PD smokers showed no difference in mortality rate between never- and ex-smokers. To our knowledge, no study has compared the causes of death according to smoking status in patients with PD. In our results, PD smokers tended to have fewer deaths from neurologic causes but were significantly more likely to die from lung cancer. Mean follow-up duration from PD onset to death was similar between groups according to smoking status. Interestingly, although initial LEDD was similar between groups, LEDD at the last follow-up was significantly lower in current smokers than never- and ex-smokers, which might suggest a neuroprotective effect of smoking on PD progression. Compared to PD never smokers, PD current smokers have lived somewhat longer with a relatively lower dose of LEDD and died more from neoplasms such as lung cancer. Based on our results, smoking seems to have a neuroprotective effect on PD mortality; however, smoking itself causes smoking-related death; thus, overall, the association between smoking and mortality in PD is nonsignificant. This could be a possible explanation for the nonsignificant association between smoking and mortality in patients with PD, which has been consistently reported in previous studies. It has not been suggested whether quitting or maintaining cigarette smoking is helpful for disease management in PD. There seems to be a complex association between mortality and neuroprotective effects or harms of smoking in PD. Based on our results, cigarette smoking, for its neuroprotective effect, is not recommended for individuals with PD. Further studies are warranted to determine the specific ingredients of cigarettes that are relevant to their neuroprotective effects.

The interpretation of analyses of the cause of death in individuals with PD needs caution. In this study, we used the death certificate and classified the cause of death into broad groups according to ICD-10 codes. Various motor and non-motor symptoms of PD, such as falls or dysphagia, could increase the risk of mortality. Therefore, the causes of death in individuals with PD are very complicated. Pneumonia has been suggested as the major cause of death in PD in previous clinical studies^38,39^, whereas PD itself was registered as the primary or underlying cause of death on the death certificate in previous studies^39,40^. In studies using death certificates, complications of PD, such as pneumonia or falls, that lead to death would tend to be regarded as deaths due to PD (neurological cause of death). Therefore, we thought it seemed difficult to evaluate the detailed cause of death of PD in this claims-based study. In this study, we tried to investigate the differences in the cause of death according to smoking status in PD, and therefore, the broad cause of death among PD groups according to smoking status was compared.

Several limitations need to be considered in the interpretation of our results. First, PD and other comorbidities were defined by diagnostic codes in the claims data from the NHIS without clinical or laboratory data; hence, we cannot exclude misclassification. Second, clinical information, such as motor symptoms or PD subtype, related to PD severity was unavailable in this claims-based cohort study. Therefore, we only included newly diagnosed PD and used LEDD as a proxy for PD severity. Third, although we tried to adjust many variables, including socioeconomic status and comorbidities, dietary habits such as caffeine or vitamin were not obtained from the NHIS database and could not be included in the analysis. Fourth, the number of heavy smokers in the PD group was too small, and the results of analyses by smoking duration and intensity should be interpreted with caution. Fifth, the cause of death was obtained from death certificate data from the Korea National Statistical Office. Future studies on the association between smoking status and PD mortality with information on the cause of death based on clinical studies are warranted. Sixth, there were significant differences in baseline characteristics according to smoking status in PD patients. Although we tried to adjust for many variables, these differences could bias the results, including cause of death. Finally, in this population-based observational study, we could not elucidate the exact mechanisms underlying the association between smoking and mortality. Based on the analysis of the cause of death, smoking seems to have neuroprotective mechanisms in PD mortality, and clinical trials are needed to confirm this mechanism.

## Methods

### Data source

The Korean NHIS, a single-payer system, is mandatory for all residents of Korea. Healthcare providers submit claims of their healthcare services to the NHIS electronically and are reimbursed for their services. The claims data of the NHIS include healthcare utilization information, such as patient demographics, diagnosis, procedures, surgical history, and prescription drugs, which are provided for research purposes. The International Classification of Diseases 10th revision (ICD-10) was adopted in the NHIS to classify diseases into diagnostic codes. The NHIS provides a biannual NHSP without cost to all beneficiaries aged ≥40 years. The NHSP includes history taking, physical examinations, anthropometric measurements, and questionnaires with anticipatory guidance. This study was approved by the Institutional Review Board of the Korea University Guro Hospital, which waived the requirement for informed consent.

### Study population

**Fig. 3:**
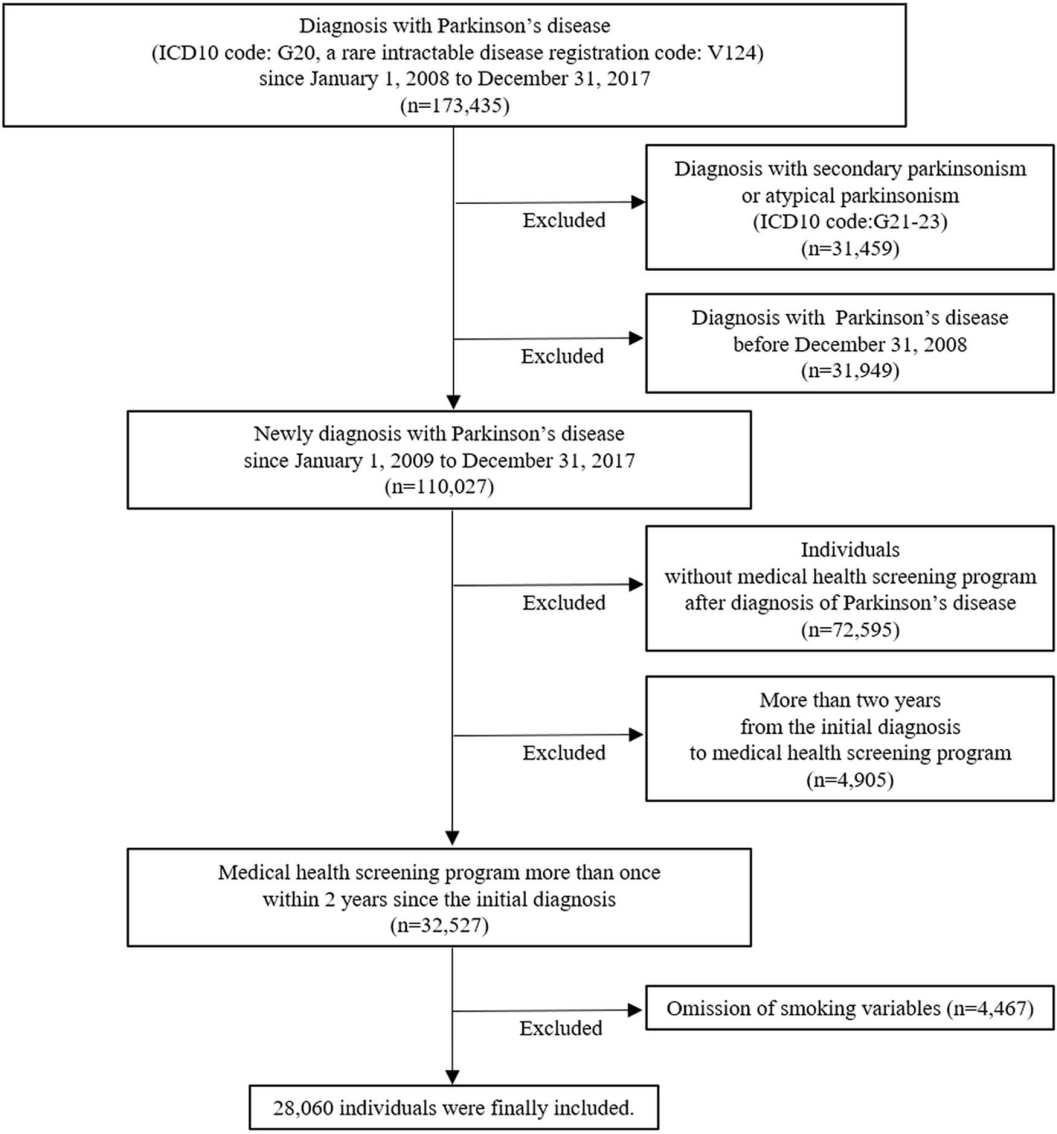
Flow chart of sample selection for cohort study.

In Korea, the government started operating a registration program for rare intractable diseases, including PD, in 2004. Among the entire nationwide population data, individuals with newly diagnosed PD (ICD-10 code: G20 and a rare intractable disease registration code: V124) between 2008 and 2017 were included, and individuals with diagnostic codes of secondary parkinsonism or atypical parkinsonism (ICD-10 code: G21–G23) were excluded. We also excluded individuals diagnosed with PD before 2009 so that only new-onset PD was included in the analysis. Among them, those who had received NHSP at least once within two years after the initial diagnosis of PD were finally selected. We longitudinally followed 28,060 individuals to investigate the association between smoking status and mortality in patients with PD (Fig. 3).

### Smoking status

All individuals in the NHSP were required to complete self-report questionnaires consisting of questions including lifestyle factors. Smoking status was categorized, based on the Centers for Disease Control and Prevention, into the following three groups: current smoker (had smoked ≥100 cigarettes in their lifetime and currently smoking), ex-smoker (had smoked ≥100 cigarettes in their lifetime, did not currently smoke), and never smoker^41^. In addition, pack years, duration, and intensity of smoking were also assessed to elucidate the association with all-cause mortality in PD. The pack-years smoked was measured by multiplying the number of packs smoked per day by the duration of smoking. Duration and intensity of smoking were measured based on responses to the following question: “How many years have you been smoking?” or “How many cigarettes have you been smoking per day?”

### Other variables

The endpoint of this study was death in individuals with PD, and all-cause mortality up to December 31, 2017, was evaluated. As there was only the date of death in the NHIS database, the cause of death was identified by linking the NHIS data with death certificate data from the Korea National Statistical Office. The causes of death were classified according to the ICD-10 code: infection (A00-B99), neurologic (G00–G99), circulatory (I00–I99), pulmonary (J00–J99), digestive (K00–K93), neoplasm (C00–D48), metabolic (E00-E99), mental (F00-F99), genitourinary (N00-N99), etc.

Demographic variables, such as age and sex, were based on the time of diagnosis. The income deciles of participants were categorized into four groups based on the NHI premium. The Charlson Comorbidity Index and other comorbidities were identified based on the corresponding diagnostic codes of the diseases. Clinical information related to PD severity was unavailable in this claims-based database. Therefore, LEDD, the sum of the total daily medications received by PD patients to relieve symptoms, was used as a proxy for PD severity.

Anthropometric factors, including height, weight, and blood pressure (systolic and diastolic), were assessed. Body mass index (BMI) was calculated as weight divided by height in meters squared (kg/m^2^). Venous samples were drawn after overnight fasting to determine fasting plasma glucose, total cholesterol, and hemoglobin levels. Alcohol consumption was categorized into two groups: (1) nondrinkers or (2) participants who consumed alcohol. Physical activity was categorized into two groups by calculating standard metabolic equivalent of task (MET) values of physical activity: (1) light intensity (<600 METs) or (2) moderate/vigorous intensity (≥600 METs)^42^.

### Statistical analysis

Analysis of variance (ANOVA) was performed for continuous variables, and the chi-squared test was performed for categorical variables to examine the characteristics of PD patients according to smoking status. Survival analysis over time was performed through a log-rank test using Kaplan‒Meier curves. We performed univariate and multivariate Cox proportional hazards analyses, including variables such as age, sex, socioeconomic status, comorbidities, alcohol consumption, physical activity, waist circumference, and LEDD. Cox proportional hazards analyses were conducted to estimate the hazard ratio (HR) and 95% confidence interval (CI), describing the relative risk of death according to smoking status. Since smoking status could change over time, we conducted a sensitivity analysis in PD patients who had had NHSP more than twice and had maintained the same smoking status on both NHSP questionnaires. All statistical analyses were performed using SAS 9.4 (SAS Institute Inc., Cary, NC, USA), and a two-sided *p* value of <0.05 was considered statistically significant. The significance level of Bonferroni post-hoc analysis was set at *p* < 0.05/3 (0.05/the total number of ANOVA models).

### Systematic literature review

A systematic review was conducted and reported in accordance with the PRISMA (Preferred Reporting Items for Systemic Reviews and Meta-Analyses) statement^43^. References on the association between smoking status and mortality of PD patients were searched in the MEDLINE (PubMed), EMBASE, and Cochrane Central Register of Controlled Trials databases up to June 2022 without any date restrictions. Key terms used to conduct the literature search were selected and combined with the following English terms and their equivalents: “Parkinson’s disease,” “smoking,” AND “mortality.” The search strategy for each database is outlined in eAppendix 1. We included studies that measured baseline smoking status and prospectively followed participants to identify mortality in patients with PD. Any relevant study, regardless of the design, was included. Literature review, expert opinions, and studies with insufficient outcome variables were excluded. The title and abstract of each article were screened for eligibility. Subsequently, full-text articles were assessed for inclusion, and data were extracted from selected studies. Owing to the small number and clinical heterogeneity of the extracted studies through a literature search, we could not pool the results in the meta-analysis. Data were analyzed using descriptive statistics.

### Reporting summary

Further information on research design is available in the Nature Research Reporting Summary linked to this article.

## Supplementary information

Reporting Summary

Supplementary materials

## Data Availability

This study was performed using the NHIS database (https://nhiss.nhis.or.kr/), but restrictions apply to the availability of these data, which were used under license for the current study, and so are not publicly available. The source NHIS data do not belong to the researchers, and we are not allowed to transfer the data files to a third party under Korean law. The data can be used after approval of the Institutional Review Board and the Korea NHIS Big Data Operations Department.

## Code availability

The underlying code for this study is not publicly available but may be made available to qualified researchers at reasonable request from the corresponding author.
