# Supplementary material for: Association between smoking and all-cause mortality in Parkinson’s disease
Source: NPJ Parkinsons Dis. 2023 Apr 11;9:59. doi: 10.1038/s41531-023-00486-0 (PMC10085986; doi:10.1038/s41531-023-00486-0)
Supplement: Supplementary file 2 — Supplementary materials [file 41531_2023_486_MOESM2_ESM.pdf]

Appendix 1. Search strategies for systematic review

PubMed

| Search | Query | Items found |
|---|---|---|
| #13 | #12 NOT (meta-analysis[Filter] OR systematic review[Filter]) | 133 |
| #12 | #11 AND humans[Filter] | 138 |
| #11 | #7 AND #10 | 154 |
| #10 | #8 OR #9 | 2,617,790 |
| #9 | survival[MeSH Terms] OR mortality[MeSH Terms] OR death[MeSH Terms] | 566,519 |
| #8 | survival[Title/Abstract] OR mortality[Title/Abstract] OR death[Title/Abstract] | 2,401,562 |
| #7 | #3 AND #6 | 1,143 |
| #6 | #4 OR #5 | 383,875 |
| #5 | tobacco[MeSH Terms] OR smoking[MeSH Terms] ORcigarette smoking[MeSH Terms] | 190,370 |
| #4 | smoking[Title/Abstract] OR cigarette[Title/Abstract] OR tobacco[Title/Abstract] OR cigar[Title/Abstract] | 333,601 |
| #3 | #1 OR #2 | 122,374 |
| #2 | Parkinson disease[MeSH Terms] | 77,008 |
| #1 | parkinson's disease[Title/Abstract] OR parkinson disease[Title/Abstract] OR pakrinson[Title/Abstract] | 108,550 |

EMBASE

| No. | Query | Results |
|---|---|---|
| #19 | #17 AND 'human'/de AND ('article'/it OR 'article in press'/it) | 134 |
| #18 | #17 AND 'human'/de AND ('article'/it OR 'article in press'/it) | 134 |
| #17 | #16 AND 'human'/de | 300 |
| #16 | #9 AND #15 | 321 |
| #15 | #10 OR #14 | 4464945 |
| #14 | #11 OR #12 OR #13 | 2867240 |

| | | |
|---|---|---|
| #13 | death'/exp | 796233 |
| #12 | mortality'/exp | 1280323 |
| #11 | survival'/exp | 1300348 |
| #10 | survival OR mortality OR death | 4263223 |
| #9 | #3 AND #8 | 2002 |
| #8 | #5 OR #6 OR #7 | 476755 |
| #7 | cigarette smoking'/exp | 60834 |
| #6 | smoking'/exp | 440833 |
| #5 | tobacco'/exp | 53616 |
| #4 | smoking OR cigarette OR tobacco OR cigar | 651108 |
| #3 | #1 OR #2 | 184518 |
| #2 | parkinson disease'/exp | 180055 |
| #1 | parkinson disease' OR pakrinson | 183651 |

Cochrane Central Register of Controlled Trials (CENTRAL)

#1 "parkinson's disease" OR "parkinson disease" OR pakrinson 11081

#2 MeSH descriptor: [Parkinson Disease] explode all trees 4703

#3 smoking OR cigarette OR tobacco OR cigar 40586

#4 MeSH descriptor: [Tobacco] explode all trees 193

#5 MeSH descriptor: [Smoking] explode all trees 6499

#6 MeSH descriptor: [Cigarette Smoking] explode all trees 163

#7 survival OR mortality OR death 225996

#8 MeSH descriptor: [Survival] explode all trees 134

#9 MeSH descriptor: [Mortality] explode all trees 14079

#10 MeSH descriptor: [Death] explode all trees 2362

#11 #1 OR #2 11081

#12 #3 OR #4 OR #5 OR #6 40587

#13 #7 OR #8 OR #9 OR #10 226515

#14 #11 AND #12 AND #13 37

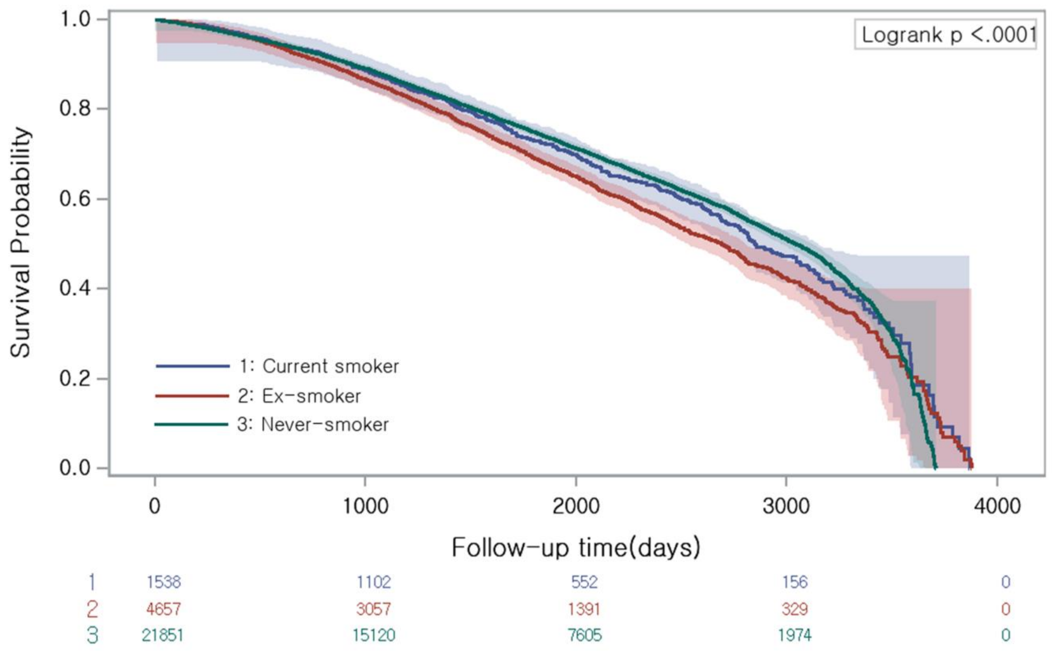


Supplementary figure 1. Cumulative proportion of all-cause mortality over time in Parkinson's disease according to smoking status. Shading represents 95% confidence intervals (Cls).

Supplementary table 1. Association of intensity and duration of smoking on the risk of all-cause mortality in individuals with Parkinson's disease

| | | PD (n) | Mortality (n) | Person-years | Mortality rate | Adjusted HR | P for trend |
|---|---|---|---|---|---|---|---|
| **Pack-years (PY)** | | | | | | | |
| Never-smoker | | 21,880 | 6,574 | 97921.41 | 67.14 | 1.00 | 0.7695 |
| Ex-smoker | < 10 | 3,940 | 1,342 | 13499.81 | 99.41 | 1.06 (0.98-1.14) | |
| | ≥10 | 713 | 230 | 5931.73 | 38.77 | 1.00 (0.91-1.09) | |
| Current smoker | < 10 | 1,226 | 427 | 4407.70 | 96.88 | 1.27 (1.13-1.43) | |
| | ≥10 | 309 | 90 | 2633.71 | 34.17 | 0.82 (0.71-0.96) | |
| **Duration of smoking (year)** | | | | | | | |
| Never-smoker | | 21,880 | 6,574 | 97921.41 | 67.14 | 1.00 | 0.7015 |
| Ex-smoker | < 10 | 3,760 | 1,294 | 13499.81 | 95.85 | 1.05 (0.97-1.14) | |
| | ≥10 | 893 | 278 | 5931.73 | 46.87 | 1.01 (0.93-1.10) | |
| Current smoker | < 10 | 1,159 | 401 | 4407.70 | 90.98 | 1.34 (1.17-1.53) | |
| | ≥10 | 376 | 116 | 2633.71 | 44.04 | 0.87 (0.76-0.99) | |
| **Intensity of smoking (Cigarette per day)** | | | | | | | |
| Never-smoker | | 21,880 | 6,574 | 97921.41 | 67.14 | 1.00 | 0.8495 |
| Ex-smoker | < 10 | 1,890 | 845 | 8793.02 | 96.10 | 1.09 (1.01-1.18) | |
| | ≥10 | 2,763 | 727 | 10638.52 | 68.34 | 0.97 (0.89-1.06) | |
| Current smoker | < 10 | 774 | 354 | 3710.63 | 95.40 | 1.19 (1.06-1.33) | |
| | ≥10 | 761 | 163 | 3330.78 | 48.94 | 0.85 (0.72-1.00) | |

Mortality rate is the incidence of mortality per 1000 person-year

Adjusted for age, sex, income level, residential area, comorbidities, lifestyle factors (drinking, physical activity), waist circumference, and levodopa equivalent daily dose (last follow-up)

Supplementary table 2. Comparison of cause of mortality according to smoking status in Parkinson's disease

| | Never-smoker | Ex-smoker | Current smoker | p value |
|---|---|---|---|---|
| Neurologic | 765 (28.22%) | 181 (27.42%) | 29 (19.21%) | 0.0572 [b,c] |
| Circulatory | 463 (17.08%) | 100 (15.15%) | 26 (17.22%) | 0.6255 |
| Pulmonary | 416 (15.34%) | 110 (16.67%) | 25 (16.56%) | 0.6695 |
| Neoplasm | 293 (10.81%) | 105 (15.91%) | 32 (21.19%) | <.0001 [a,b] |
| Digestive | 80 (2.95%) | 21 (3.18%) | 3 (1.99%) | 0.806 |
| Others | 694 (25.6%) | 143 (21.67%) | 36 (23.84%) | 0.1775 |

P for Bonferroni post-hoc analysis < 0.015; a:Never-smoker vs Ex-smoker, b:Never-smoker vs Current smoker and c:Ex-smoker vs Current smoker.

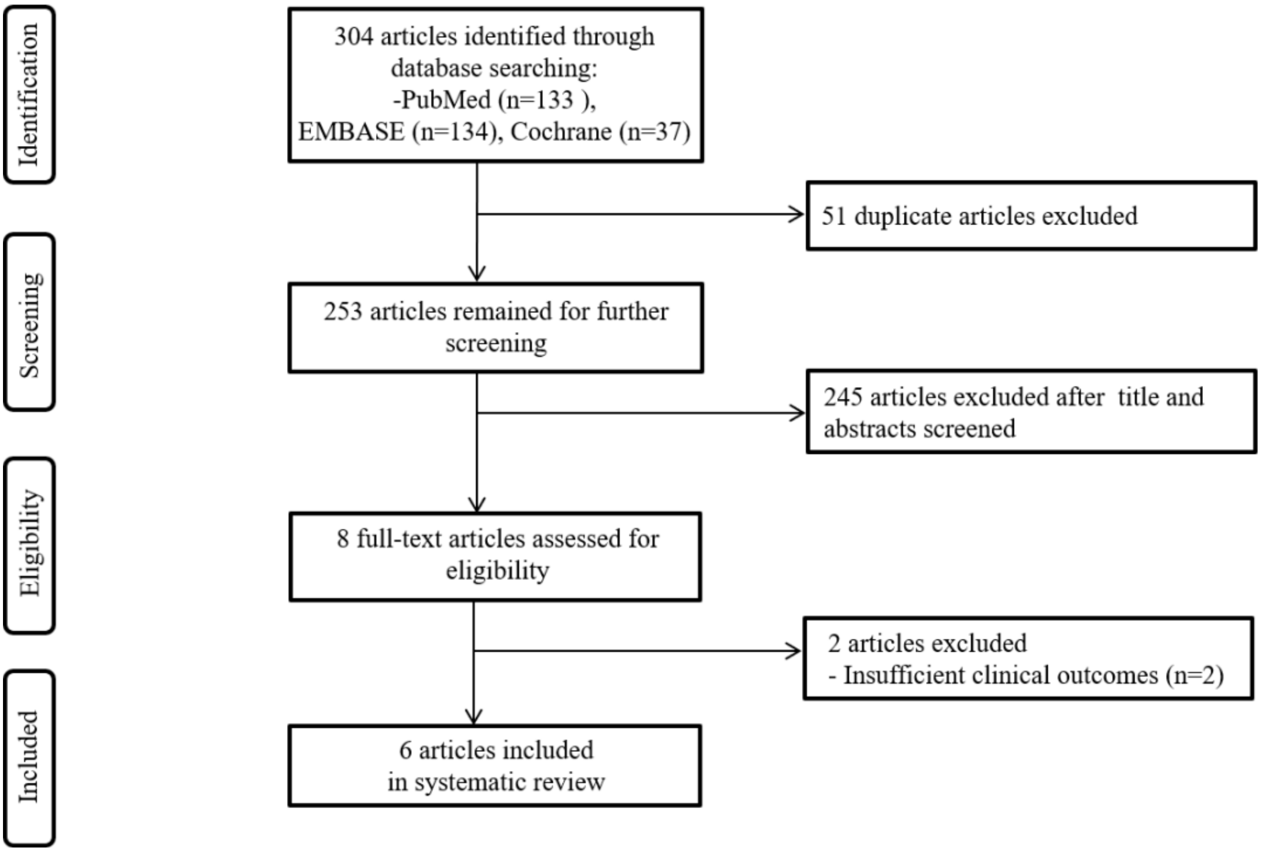


Supplementary figure 2. Flow chart of study selection for systematic review
